# Breast Cancer Stage Among Ukrainian Refugees in Poland

**DOI:** 10.1001/jamanetworkopen.2025.6215

**Published:** 2025-04-22

**Authors:** Jakub Skórniak, Lukasz Rabalski, Bartłomiej Szynglarewicz, Bartosz Dołęga-Kozierowski, Piotr Kasprzak, Marcin Ziętek, Adam Maciejczyk, Edyta Pawlak, Martyna Krejmer-Rabalska, Katarzyna Skuza, Rafał Matkowski

**Affiliations:** 1 Breast Unit, Wroclaw Comprehensive Cancer Centre, Wroclaw, Poland; 2 Biological Threats Identification and Countermeasure Centre, Military Institute of Hygiene and Epidemiology, Pulawy, Poland; 3 Department of Virus Molecular Biology, Intercollegiate Faculty of Biotechnology, University of Gdansk and Medical University of Gdansk, Gdansk, Poland; 4 Department of Oncology, Faculty of Medicine, Wroclaw Medical University, Wroclaw, Poland; 5 Laboratory of Immunopathology, Department of Experimental Therapy, Hirszfeld Institute of Immunology and Experimental Therapy, Polish Academy of Sciences, Wroclaw, Poland; 6 4th Military Clinical Hospital with Polyclinic SPZOZ, Wrocław, Poland; 7 Department of Recombinant Vaccines, Intercollegiate Faculty of Biotechnology, University of Gdansk and Medical University of Gdansk, Gdansk, Poland

## Abstract

**Importance:**

Millions of Ukrainians displaced by war have sought refuge in Poland, posing challenges for cancer care. Determining whether these refugees present with more-advanced breast cancer is crucial for guiding resource allocation.

**Objective:**

To evaluate the associations between Ukrainian refugee status and clinical presentation of breast cancer compared with Ukrainian permanent residents in Poland and the general Polish population.

**Design, Setting, and Participants:**

This retrospective cohort study was conducted between February 2021 and February 2024 at a single tertiary oncology center (Lower Silesian Oncology, Pulmonology, and Hematology Centre, Wrocław, Poland) and included adult women with a diagnosis of stage 0 to IV breast cancer, regardless of treatment intent. Participants were categorized as Ukrainian permanent residents (UPRs), Ukrainian war refugees (UWRs), or general Polish population (GPP).

**Exposures:**

War refugee status (UWR) vs permanent residence (UPR) vs general Polish population (GPP) and a diagnosis of breast cancer.

**Main Outcomes and Measures:**

Primary outcomes were clinical stage (0-IV) at diagnosis, tumor grade (G1-G3), and age at presentation. Stage distributions and odds ratios (ORs) were evaluated using weighted logistic regression, adjusting for age and time period (before the war, first war year, and second war year).

**Results:**

Of 3259 patients, 44 were in the UWR group (mean [SD] age, 52.9 [13.5] years), 72 were in the UPR group (mean [SD] age, 49.9 [11.4] years), and 3143 were in the GPP group (mean [SD] age, 59.5 [12.6] years). Stage III to IV disease occurred in 19 patients (43.2%) in the UWR group vs 849 patients (27.0%) in the GPP group. Adjusted analysis showed that refugees had a 2.00-fold increased odds (95% CI, 1.06-3.76; *P* = .03) of stage III to IV disease and 2.42-fold increased odds (95% CI, 1.29-4.55; *P* = .006) of grade 3 tumors.

**Conclusions and Relevance:**

In this retrospective cohort study, war refugee status was associated with a higher prevalence of aggressive tumor grades and more-advanced stage disease at diagnosis. Although aggressive breast cancer subtypes are more common in younger women, the disproportionate burden of advanced disease in this cohort highlights the impact of health care access barriers and delayed presentation. These findings underscore the need for tailored strategies, such as accessible screening and early diagnostic interventions, to improve outcomes among Ukrainian refugees in Poland.

## Key Points

**Question:**

What are the age and stage disparities in breast cancer presentation between Ukrainian refugees, Ukrainian permanent residents, and the general Polish population?

**Findings:**

In this cohort study of 3259 women treated at a single cancer center in Poland from 2021 to 2024, Ukrainian refugees were younger yet had 2.00-fold higher odds of advanced-stage (III-IV) disease and 2.42-fold higher odds of grade 3 tumors compared with the general Polish population.

**Meaning:**

These results suggest that refugee status, not age alone, may lead to delayed diagnosis, highlighting the need for focused early detection and health care support.

## Teaser

This cohort study evaluates the clinical presentation of breast cancer among Ukrainian refugees in Poland compared with Ukrainian permanent residents and the general Polish population.

## Introduction

The Russian invasion of Ukraine in February 2022 precipitated a humanitarian crisis, displacing millions of Ukrainians. By late 2023, nearly 4 million Ukrainian refugees had sought shelter in European Union countries, with Poland alone accommodating approximately 1 million.^1^ This sudden influx has posed unprecedented challenges for Poland’s health care system, particularly in oncology.^2,3,4,5^

Breast cancer—the most prevalent malignant entity among women globally—has seen a pronounced increase in cases among Ukrainian refugees seeking care in Poland.^6^ Data from the World Health Organization and the National Cancer Registry indicate that in 2020, 12 824 cases of breast cancer were reported among Ukrainian women, with 4998 related deaths.^7^ The ongoing conflict has further complicated access to oncologic services, frequently causing diagnostic and treatment delays.^8^

Studies of other conflict-driven migrations suggest that forced displacement consistently leads to more-advanced disease at presentation and inferior outcomes. Research on Syrian refugees in Turkey and Jordan demonstrated that refugees often had later-stage cancers and encountered major barriers to prompt diagnosis and therapy, including lack of health care access, delayed referrals, and the stress of displacement.^9,10,11^ In Poland, although the government’s rapid extension of comprehensive public health services for Ukrainian refugees helped address immediate needs, it strained already limited oncologic resources.^12,13^

This study compares breast cancer presentation in 3 groups: Ukrainian permanent residents (UPRs), newly arrived Ukrainian war refugees (UWRs), and the general Polish population (GPP) to evaluate how refugee status and ongoing wartime disruption have affected clinical stage, tumor biology, and diagnosis timing. Our findings may inform health care policy decisions and resource allocation for cancer care in humanitarian crises.

## Methods

### Study Design

This retrospective cohort study followed the Strengthening the Reporting of Observational Studies in Epidemiology (STROBE) reporting guidelines. The study was approved by the Bioethical Committee of the Medical University of Wroclaw. A waiver of informed consent was granted by the Bioethical Committee owing to the retrospective design of the study. We analyzed data from patients treated between February 2021 and February 2024, encompassing 3 distinct periods: before the war (period 1, February 2021-February 2022), first war year (period 2, February 2022-February 2023), and second war year (period 3, February 2023-February 2024).

### Setting and Participants

The study was conducted at the Lower Silesian Oncology, Pulmonology, and Hematology Centre in Wrocław, Poland, a tertiary oncology center serving the Lower Silesian region with a population of approximately 2.9 million inhabitants. Eligible participants were adult women (aged ≥18 years) with a breast cancer diagnosis (stage 0-IV) who presented to our tertiary oncology center between February 2021 and February 2024, regardless of treatment intent. Men were excluded. Although many patients underwent breast surgery (curative or palliative), the study population was not limited to surgical cases only; all patients treated at our center during the specified period were included, including those with stage IV disease. On the basis of nationality and residency documentation, participants were categorized into 3 groups: UPR, defined as Ukrainian nationals residing in Poland before February 2022; UWR, defined as Ukrainian nationals arriving in Poland as refugees after February 2022; and the GPP. UPRs were defined as individuals who had been living, working, and insured in Poland for at least 1 year before the outbreak of war. These individuals were proficient in Polish and had established residency, distinguishing them from newly arrived war refugees.

### Data Sources and Collection

We extracted clinical and pathological information from medical records, including age at surgical intervention, clinical tumor size (cT), nodal status (cN), metastasis status (cM), overall cancer stage (0-IV), histological grade (G1-G3), and cancer type (ductal carcinoma in situ, invasive carcinoma of no special type, invasive lobular carcinoma, or other). Ukrainian nationality and refugee status were verified through official documentation of date of entry to Poland and self-reported residency status.

### Main Outcomes and Measures

The primary outcomes were clinical stage at diagnosis (categorized as 0-IV according to TNM classification), tumor grade (G1-G3), and age at presentation. We also analyzed the distribution of tumor characteristics, including clinical T stage, N stage, and histological type.

### Statistical Analysis

We compared demographic and clinical variables across groups using 1-way analysis of variance for continuous data (age) and χ^2^ tests for categorical variables (stage, grade, and cancer type). We conducted pairwise *t* tests with Bonferroni correction to assess differences in age between groups. To address sample-size discrepancies, we performed weighted logistic regression analyses with refugee status (UWR or UPR vs GPP) as the primary variable, adjusting for age and time period. Results are reported as odds ratios (ORs) with 95% CIs. A 2-sided *P* < .05 was considered statistically significant. All analyses were performed using Stata statistical software version 18.0 (StataCorp).

Missing data accounted for less than 10% across all variables and were handled through complete case analysis. The number of available observations is reported for each analysis.

## Results

### Patient Characteristics

Among 3259 women who met eligibility criteria and had received a diagnosis of or had been treated for breast cancer between February 2021 and February 2024, 72 were in the UPR group, 44 were in the UWR group, and 3143 were from the GPP. The mean (SD) age at diagnosis was 49.9 (11.4) years for the UPR group, 52.9 (13.5) years for the UWR group, and 59.5 (12.6) years for the GPP group (Table 1). Both Ukrainian groups were significantly younger than the GPP group, but no significant age difference was observed between the UPR and UWR groups.

**Table 1.** Baseline Characteristics of Study Participants by Group

Characteristic	Patients, No. (%)			*P* value
	Ukrainian permanent residents (n = 72)	Ukrainian war refugees (n = 44)	General Polish population (n = 3143)	
Age, mean (SD), y	49.9 (11.4)	52.9 (13.5)	59.5 (12.6)	<.001
Study period				
Period 1^a^	16 (22.2)	0	994 (31.6)	<.001
Period 2^b^	23 (31.9)	29 (65.9)	1114 (35.4)	
Period 3^c^	33 (45.8)	15 (34.1)	1035 (32.9)	

^a^ Refers to February 2021 to February 2022, before the war.

^b^ Refers to February 2022 to February 2023, the first war year.

^c^ Refers to February 2023 to February 2024, the second war year.

The distribution of patient characteristics across the 3 study periods differed significantly (χ^2^ = 26.8; *P* < .001). Before the Russian invasion (period 1), there were 16 UPR (22.2%) and 994 GPP (31.6%) patients, with no UWR as expected. During the first year of war (period 2), there were 23 UPR (31.9%), 29 UWR (65.9%), and 1114 GPP (35.4%) patients. In the second year (period 3), there were 33 UPR (45.8%), 15 UWR (34.1%), and 1035 GPP (32.9%) patients (Table 1).

### Cancer Stage and Grade

Analysis of combined data from all periods showed that UWR patients presented with more-advanced disease compared with both UPR and GPP patients (Table 2). Stage III to IV disease was present in 19 patients (43.2%) in the UWR group compared with 13 patients (18.0%) in the UPR group, and 849 patients (27.0%) in the GPP group. Similarly, grade 3 tumors were more frequent among UWR (15 patients [34.1%]) compared with UPR (9 patients [12.5%]) and GPP (676 patients [21.5%]) patients.

**Table 2.** Cancer Characteristics by Ukrainian Residency Status

Characteristic	Patients, No. (%)		*P* value
	Ukrainian permanent residents (n = 72)	Ukrainian war refugees (n = 44)	
Tumor size			
T0-2	58 (80.6)	27 (61.4)	.09
T3-4	14 (19.4)	17 (38.6)	
Nodal status			
N0	49 (68.1)	18 (40.9)	.02
N+	23 (31.9)	26 (59.1)	
Stage			
0-II	59 (82.0)	25 (56.8)	.03
III-IV	13 (18.0)	19 (43.2)	
Grade			
G1-2	63 (87.5)	29 (65.9)	.002
G3	9 (12.5)	15 (34.1)	

Tumor phenotype data, including histologic subtypes and tumor grades, were analyzed and are summarized within the study cohorts. The distribution of aggressive tumor phenotypes, such as high-grade no special type and ductal carcinoma in situ nuclear grade 3, was higher among UWR patients, supporting the hypothesis that these patients present with biologically aggressive disease.

Weighted logistic regression analyses, adjusting for age and time period, confirmed these findings (Table 3). Compared with GPP patients, UWR patients had significantly lower odds of presenting with stage 0 to I disease (OR, 0.43; 95% CI, 0.23-0.80; *P* = .009) and higher odds of stage III to IV disease (OR, 2.00; 95% CI, 1.06-3.76; *P* = .03). UWR patients also demonstrated higher odds of grade 3 tumors (OR, 2.42; 95% CI, 1.29-4.55; *P* = .006). In contrast, UPR patients showed no significant differences from GPP in stage distribution but had lower odds of high-grade tumors (OR for grade 3, 0.67; 95% CI, 0.33-1.35; *P* = .26).

**Table 3.** Analysis of Cancer Characteristics Among Study Groups^a^

Characteristic	Ukrainian permanent residents vs general Polish population		Ukrainian war refugees vs general Polish population	
	OR (95% CI)	*P* value	OR (95% CI)	*P* value
Stage				
0-I	0.91 (0.54-1.54)	.73	0.43 (0.23-0.80)	.009
II	1.13 (0.69-1.87)	.62	1.08 (0.58-2.01)	.81
III-IV	0.72 (0.39-1.33)	.30	2.00 (1.06-3.76)	.03
Grade				
G1	2.66 (1.62-4.38)	<.001	0.53 (0.19-1.50)	.23
G2	0.59 (0.37-0.95)	.03	0.66 (0.36-1.21)	.18
G3	0.67 (0.33-1.35)	.26	2.42 (1.29-4.55)	.006

Abbreviation: OR, odds ratio.

^a^ Results are based on weighted logistic regression to account for sample size differences between groups.

### Time Period Analysis

Analyses stratified by time period showed consistent patterns. In both period 2 and period 3, UWR maintained higher proportions of advanced-stage and high-grade disease compared with UPR and GPP groups, although some comparisons did not reach statistical significance owing to smaller subgroup sizes. The proportion of stage III to IV disease among UWR patients was 41.4% in period 2 and 46.7% in period 3, compared with 17.4% and 18.2% for UPR patients and 26.8% and 27.3% for GPP patients, respectively.

## Discussion

This retrospective cohort study found that UWR patients presented with more-advanced breast cancer compared with both UPR and GPP patients. Despite being significantly younger than GPP patients, the UWR group had twice the odds of presenting with stage III to IV disease and 2.4 times higher odds of grade 3 tumors. These findings were observed consistently across the first 2 years following the Russian invasion of Ukraine, suggesting a persistent pattern rather than a temporary phenomenon.

The striking aspect of our findings is the markedly higher percentage of advanced-stage cancers among refugees compared with both comparison groups, despite the refugee population being approximately 7 to 10 years younger on average. This age difference enhances the importance of our findings. In well-resourced health care settings, younger age does not typically correlate with more-advanced stages at diagnosis, as many patients undergo timely clinical evaluation. However, in populations with limited access to screening and diagnostic services, such as war refugees, delays in care may result in later-stage presentation, even among younger patients. In addition, in the GPP group, mammographic screening may contribute to an older median age at diagnosis by facilitating earlier detection in women aged 50 to 69 years. The higher proportion of grade 3 tumors among refugees further suggests that delayed diagnosis and medical intervention may influence tumor progression, although differences in pathological assessment between Ukrainian and Polish centers must be considered.

In Poland, Ukrainian refugees receive oncologic treatment under the same conditions as Polish citizens, with no differences in access to medical services, reimbursement, or standard treatment protocols. Moreover, they benefit from additional support, including interpreters, coordinators, and social workers, to assist with health care navigation. Barriers such as transportation costs have also been addressed through exemptions from public transportation and highway fees. Given these provisions, treatment disparities based on refugee status are unlikely. However, future studies should investigate long-term adherence and treatment outcomes in this population.

It is important to recognize that differences in screening exposure and the natural distribution of breast cancer subtypes between the study groups may have influenced our findings. The GPP group likely includes a subset of screen-detected cases, whereas war refugees, who may have lacked access to systematic screening, were more likely to present with symptomatic disease. In addition, given that biologically aggressive breast cancer subtypes are more prevalent in younger women, the younger age at diagnosis among refugees may, in part, reflect the natural distribution of tumor biology in an unscreened population rather than solely the impact of diagnostic delays. However, the disproportionately high burden of late-stage disease in this cohort suggests that barriers to health care access and delayed symptom recognition also played an important role.

Our results align with previous studies of cancer care in refugee populations. Studies of Syrian refugees in Turkey and Jordan have consistently shown that forced displacement is associated with later-stage diagnoses and worse prognoses.^9,10^ These disparities have been attributed to limited health care access, language barriers, and displacement-related trauma.^11^ Our study extends these findings to the Ukrainian refugee population in Poland, demonstrating that even in a setting with rapid extension of public health services to refugees, substantial disparities persist.^12^

Several factors may contribute to these disparities. First, war-related disruptions likely affected health care facilities in Ukraine, leading to missed screenings and delayed diagnoses. Second, the stress of conflict and displacement may cause individuals to postpone seeking medical attention for concerning symptoms.^14^ Third, refugees entering Poland face multiple barriers, including unfamiliarity with available health care services, language difficulties, and competing priorities such as securing housing and employment.^15^

### Limitations

Despite providing valuable insights, this study faces several constraints. First, the relatively small number of war refugees in our cohort is a limitation of this study. However, this reflects the actual number of UWR patients with breast cancer and treated for it in our institution during the study period. Despite the small sample size, our findings align with existing literature on health care disparities among refugee populations, suggesting that barriers to timely diagnosis may contribute to more-advanced stage disease at presentation. Second, our study was conducted at a single tertiary oncology center, which may limit generalizability. However, this institution serves as a major referral center in the region, and our dataset includes all UWR patients treated for breast cancer during the study period. Although national data would provide additional insights, such data are not yet publicly available. Third, we lacked comprehensive data on several potentially important confounding factors, including socioeconomic status, educational level, premigration health care access and practices, and detailed comorbidity profiles. These factors might influence both the timing of initial presentation and the stage at diagnosis. Fourth, the significant age difference between populations represents a potential limitation. Although our findings remain statistically robust after age adjustment, future research would benefit from age-matched cohorts or more detailed age-stratified analyses.

## Conclusions

In this retrospective cohort study of breast cancer presentations in Poland, refugee status was associated with more-advanced disease at diagnosis and higher-grade tumors, despite a younger mean age at presentation. These findings suggest that refugee status, rather than age alone, contributes to delayed diagnosis and more-aggressive disease presentation. The combination of younger age at presentation with more-advanced disease suggests that current age-based screening recommendations may need modification for refugee populations. Health care practitioners should maintain a high index of suspicion for breast cancer in younger refugee women, and access to diagnostic services should be prioritized regardless of age. These findings highlight the need for targeted health care interventions for refugee populations, including enhanced screening programs and expedited diagnostic pathways.

## Notes

Supplement.
Data Sharing Statement
