# Supplementary material for: Breast Cancer Stage Among Ukrainian Refugees in Poland
Source: JAMA Netw Open. 2025 Apr 22;8(4):e256215. doi: 10.1001/jamanetworkopen.2025.6215 (PMC12015669; doi:10.1001/jamanetworkopen.2025.6215)
Supplement: Supplement. — Data Sharing Statement [file jamanetwopen-e256215-s001.pdf]

Data Sharing Statement

Skórniak. Breast Cancer Stage Among Ukrainian Refugees in Poland. *JAMA Netw Open*. Published April 22, 2025. doi:10.1001/jamanetworkopen.2025.6215

Data
Data available: Yes
Data types: Deidentified participant data
How to access data: lukasz.rabalski@outlook.com
When available: With publication

Supporting Documents
Document types: None

Additional Information
Who can access the data: : Deidentified patient data used in this study will be made available upon reasonable request to the corresponding author, subject to institutional data sharing regulations and privacy protections
Types of analyses: for any purpose
Mechanisms of data availability: with a signed data access agreement
